# Supplementary material for: The subthalamic nucleus contributes causally to perceptual decision-making in monkeys
Source: eLife. 2024 Sep 23;13:RP98345. doi: 10.7554/eLife.98345 (PMC11419670; doi:10.7554/eLife.98345)
Supplement: Supplementary file 1. — p-Values are raw values from Pearson correlation, not corrected for multiple testing. [file elife-98345-supp1.docx]

**Supplementary File 1. Indices of motivational state did not correlate with microstimulation effects.** P values are raw values from Pearson correlation, not corrected for multiple testing.

| **DDM components** | **Fixation Break** | | **Error Rate** | | **Mean RT** | |
| --- | --- | --- | --- | --- | --- | --- |
|  | Correlation | p Value | Correlation | p Value | Correlation | p Value |
| B_d | -0.018 | 0.897 | 0.098 | 0.481 | 0.134 | 0.335 |
| B_alpha | -0.070 | 0.615 | 0.075 | 0.591 | -0.314 | 0.021 |
| a | -0.049 | 0.723 | -0.046 | 0.739 | 0.013 | 0.927 |
| k | 0.236 | 0.085 | 0.149 | 0.283 | 0.223 | 0.105 |
| me | 0.057 | 0.680 | -0.027 | 0.844 | 0.035 | 0.802 |
| z | -0.260 | 0.057 | -0.031 | 0.822 | 0.041 | 0.768 |
| T0_ipsi | 0.205 | 0.138 | 0.049 | 0.724 | 0.166 | 0.231 |
| T0_contra | 0.060 | 0.669 | -0.010 | 0.941 | 0.270 | 0.048 |
